# Opto-thermophoretic fiber tweezers

**DOI:** 10.1515/nanoph-2018-0226

**Published:** 2019-02-12

**Authors:** Abhay Kotnala, Yuebing Zheng

**Affiliations:** Department of Mechanical Engineering, Materials Science and Engineering Program and Texas Materials Institute, The University of Texas at Austin, Austin, TX 78712, USA.; Department of Mechanical Engineering, Materials Science and Engineering Program and Texas Materials Institute, The University of Texas at Austin, Austin, TX 78712, USA

**Keywords:** thermophoresis, optical tweezers, nanoparticle trapping

## Abstract

Recent advances in opto-thermophoretic tweezers open new avenues for low-power trapping and manipulation of nanoparticles with potential applications in colloidal assembly, nanomanufacturing, life sciences, and nanomedicine. However, to fully exploit the opto-thermophoretic tweezers for widespread applications, the enhancement of their versatility in nanoparticle manipulations is pivotal. For this purpose, we translate our newly developed opto-thermophoretic tweezers onto an optical fiber platform known as opto-thermophoretic fiber tweezers (OTFT). We have demonstrated the applications of OTFT as a nanoparticle concentrator, as a nanopipette for single particle delivery, and as a nanoprobe. The simple setup and functional versatility of OTFT would encourage its use in various fields such as additive manufacturing, single nanoparticle-cell interactions, and biosensing.

## 1 Introduction

Opto-thermophoretic tweezers have emerged as a new class of tweezers for trapping and manipulation of particles [1–7]. By using either the entropy-driven forces arising from the permittivity gradient at the particle-solvent interface [8] or thermophoresis-induced electric field in a strong temperature gradient [9], particles of varying sizes (from nanometers to microns), shapes (spherical, triangular), and materials (dielectric, semiconductor, metallic, and biological) have been investigated [6, 7, 10]. The use of thermal gradient-induced forces by opto-thermophoretic tweezers offers some unique advantages as compared to the optical gradient forces used in conventional optical tweezers, both near-field [11–13] and far-field [14, 15]. The first advantage is their low-power operation, which is nearly three orders of magnitude less compared to that of conventional optical tweezers [16]. This makes them ideal for biological applications where the use of low power is critical to reduce the probability of damage to the biological particles or biomolecules, such as proteins and DNA attached to a nanoparticle. The second advantage is the versatility in trapping nanoparticles. Since the thermophoretic forces do not depend on the refractive index contrast between the nanoparticle and the surrounding medium or scale directly with the size of particle, like the optical gradient force, opto-thermophoretic tweezers have been successful in trapping yeast cells, bacteria [16], and lipid vesicles [17], which have almost no refractive index contrast with the surrounding medium, without increasing the trapping laser power. Also, metallic nanoparticles as small as 20 nm could be trapped and manipulated [9], which are otherwise difficult to trap using traditional optical tweezers. Lastly, another advantage is their simple design and fabrication. A simple plasmonic substrate, such as gold nanoislands (AuNIs) on a glass substrate capable of efficiently converting photons to phonons and producing a localized temperature gradient, can be used for their operation [18, 19]. Opto-thermophoretic tweezers unique features such as simple design, lower-power operation, and the ability to trap and manipulate particles over a wide range of sizes, shapes, and materials make them an attractive alternative to the conventional optical tweezers and provide great potential for applications in material science, biology, nanomanufacturing, colloidal science, etc. However, most of the studies so far have been limited to understanding their trapping mechanism or simple demonstrations of particle manipulation [20–23], sorting [24], colloidal assembly [25, 26], or nanoparticle printing [27]. To further develop opto-thermophoretic tweezers into a universal tool and unlock its potential for use by researchers working in diverse fields, the tweezers need to be made more simple, cheap, robust, multifunctional, integrable with other technologies, and easy to operate.

To achieve this, we transfer our opto-thermophoretic tweezers to an optical fiber platform that replaces the optical components such as mirror and objective lenses used in the traditional opto-thermophoretic tweezers, making it simple, cheaper, and alignment-free. The mature optical fiber technology would further advance its capabilities and its integration with other technologies. In addition, it would also add the capability of three-dimensional (3D) manipulation of nanoparticles, which has been currently limited to two dimensions only. Optical fiber tweezers (OFT) have been developed and used extensively for trapping and manipulation of particles [28–30], along with applications in cell patterning [31], bacteria labeling [32], and bionanophotonics [33, 34]. Some of their limitations, such as complex design and fabrication, need to be overcome for further applications of OFT. For example, OFT require precise tapering of the fiber [35, 36] or fabrication of lenses at the tip of the fiber to produce a highly focused laser beam [37, 38]. For cases not requiring focused laser beam, two optical fibers have been used, but precise alignment becomes a necessity and therefore increases the complexity of the experimental setup [39, 40]. Also, trapping of sub-500-nm nanoparticles using OFT has been limited. This problem has been overcome by using near-field OFT, but the fabrication of plasmonic nanostructures such as double nanoholes [41] and bowtie [42] at the core of the fiber tip requires complex fabrication tools like the focused ion beam or electron beam lithography, making its fabrication expensive and complex. Another major issue is the poor coupling of optical power from the fiber mode to the plasmonic nanostructures. Further modifications of the structure are needed to improve the power coupling efficiency, which further complicates its design and fabrication [43].

Here, we show that opto-thermophoretic tweezers relax the above requirements of complex design, fabrication, and efficient power coupling, making their translation to the optical fiber platform much simpler compared to the conventional optical tweezers. We show the design, fabrication, and operation of opto-thermophoretic fiber tweezers (OTFT) and use it for the 3D manipulation of a single nanoparticle. In addition, we also demonstrate some potential applications where OTFT could be particularly useful, such as in nanoparticle concentration, precise delivery of nanoparticles to cells, or as a nanoprobe. OTFT would provide a very simple platform for use by a wider research community in the field of cellular biology, material science, pharmacy, nanomanufacturing, colloidal science, and nanomedicine etc.

## 2 Results and discussion

### 2.1 OTFT design and working principle

**Figure 1:**
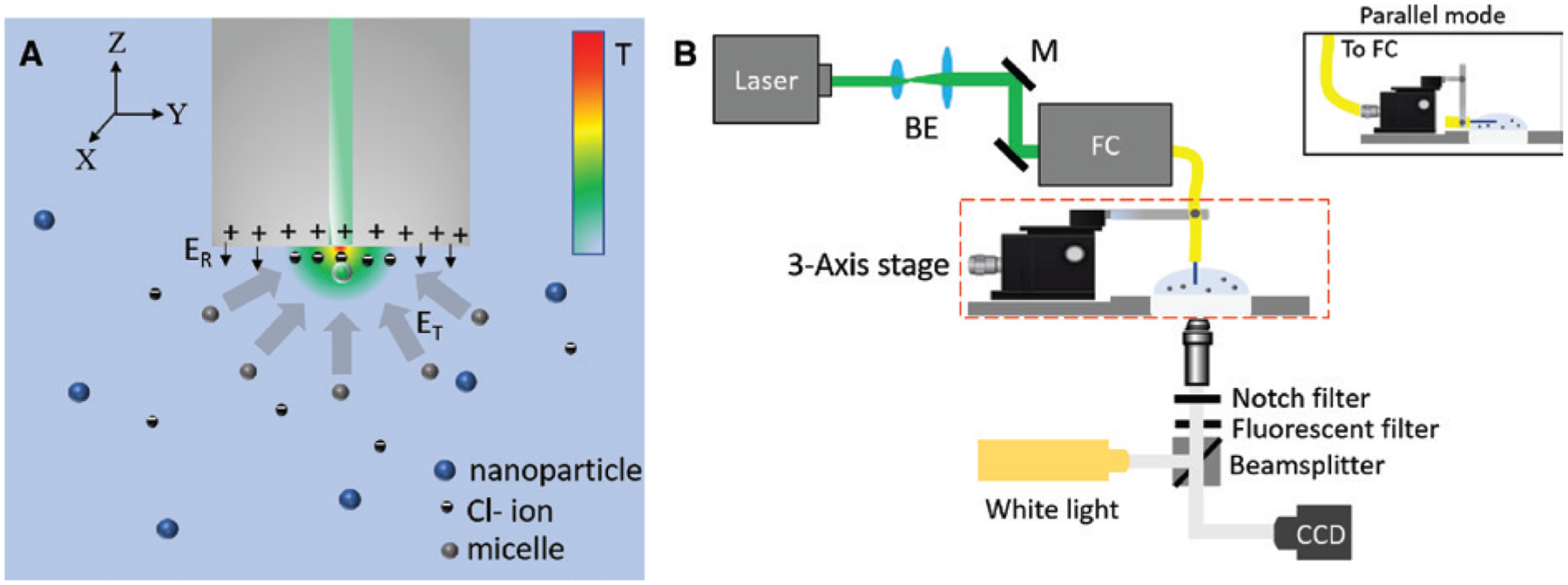
OTFT design, working principle and experimental set-up. (A) Schematic of the OTFT immersed in the trapping solution consisting of micellar ions, counterions, and the nanoparticles to be trapped. A temperature distribution is embedded in the schematic to show the temperature gradient created due to the thermoplasmonic fiber tip. (B) Schematic showing the experimental setup for trapping and manipulation of nanoparticles in 3D using OTFT. The red dashed box shows the arrangement of OTFT in normal mode. For parallel mode operation, the set up in red dashed box is replaced with the arrangement shown in the figure inset. BE, beam expander; M, mirror; FC, fiber coupler; CCD, charge coupled device.

The proposed OTFT schematic is shown in Figure 1A. It consists of a single mode fiber (SMF) with a tip, which is a porous Au film (AuNIs) fabricated simply by depositing a thin layer of gold on the fiber tip, followed by thermal annealing (see Methods section for the fabrication procedure). The thermoplasmonic fiber tip efficiently converts the photons to phonons, resulting in the formation of an optically controlled temperature gradient field as shown in Figure 1A.

To enable opto-thermoelectric forces, a cationic surfactant, cetyltrimethylammonium chloride (CTAC), is added to the nanoparticle solution to be used for trapping. CTAC molecules adsorb on the particle surface and form a positively charged molecular double layer [44] and simultaneously self-assemble into micelles when above the critical micelle concentration (0.13–0.16 mm). The CTAC micelles act as macrocations (known as micellar ions) having a high charge density and nanoscale size, while the Cl^−^ molecules act as counterions. With no light launched from the fiber, the micellar ions, counterions, and the nanoparticle are randomly dispersed in the solution. When light is launched from the fiber, the temperature gradient field generated at the tip of the fiber results in the thermophoresis of the micellar ions and Cl^−^ ions. Both the CTAC micelles and Cl^−^ ions have positive Soret coefficients and therefore undergo thermophoresis, migrating from a hot to a cold region. However, CTAC micelles have a larger Soret coefficient than Cl^−^ ions. As a result, CTAC micelles build a larger concentration gradient than Cl^−^ ions do, which leads to their spatial separation. In other words, the CTAC micelles move to the cold region while Cl^−^ ions stay close to the hot region (i.e. fiber surface as shown in Figure 1A). In steady state, this leads to the redistribution of the CTAC micelles and Cl^−^ ions generating an electric field given by [45]

$$E_{T}=\frac{k_{B}T\nabla T}{e}\frac{\sum_{i}Z_{i}n_{i}S_{Ti}}{\sum_{i}Z_{i}^{2}n_{i}},$$

where *i* indicates the ionic species (CTAC micellar ions or Cl^−^ ions), *k*_*B*_ is the Boltzmann constant, *T* is the environmental temperature, Δ*T* is the temperature gradient, *e* is the elemental charge, and *Z*_*i*_, *n*_*i*_, and *S*_*Ti*_ are the charge number, the concentration, and the Soret coefficient of *i* species, respectively. Due to CTAC micelle having a higher molecular mass and a larger Soret coefficient than the Cl^−^ ions (*S*_*T*_ [micelle] ~10^−2^ K^−1^ > *S*_*T*_ [Cl^−^] ~7.18 × 10^−4^ K^−1^), we obtain an electric field *E*_*T*_ pointing toward the fiber tip arising from the spatial redistribution of both the CTAC micelles and the Cl^−^ ions [46], which can trap the positively charged nanoparticle at the fiber core, as shown in Figure 1A. The in-plane and out-of-plane trapping forces are given by *F*_*T*_ = *qE*_*T*_. This trapping electric field is balanced by the repulsive electric field, *E*_*R*_, arising from the positive charges of the thermoplasmonic fiber tip, which is also coated by the CTAC double layers, as shown by arrows in Figure 1A. The thermo-electric field used for trapping nanoparticle in OTFT relaxes the stringent optical requirement of a highly focused laser beam needed in conventional gradient based OFT.

### 2.2 3D manipulation of nanoparticles using OTFT

**Figure 2:**
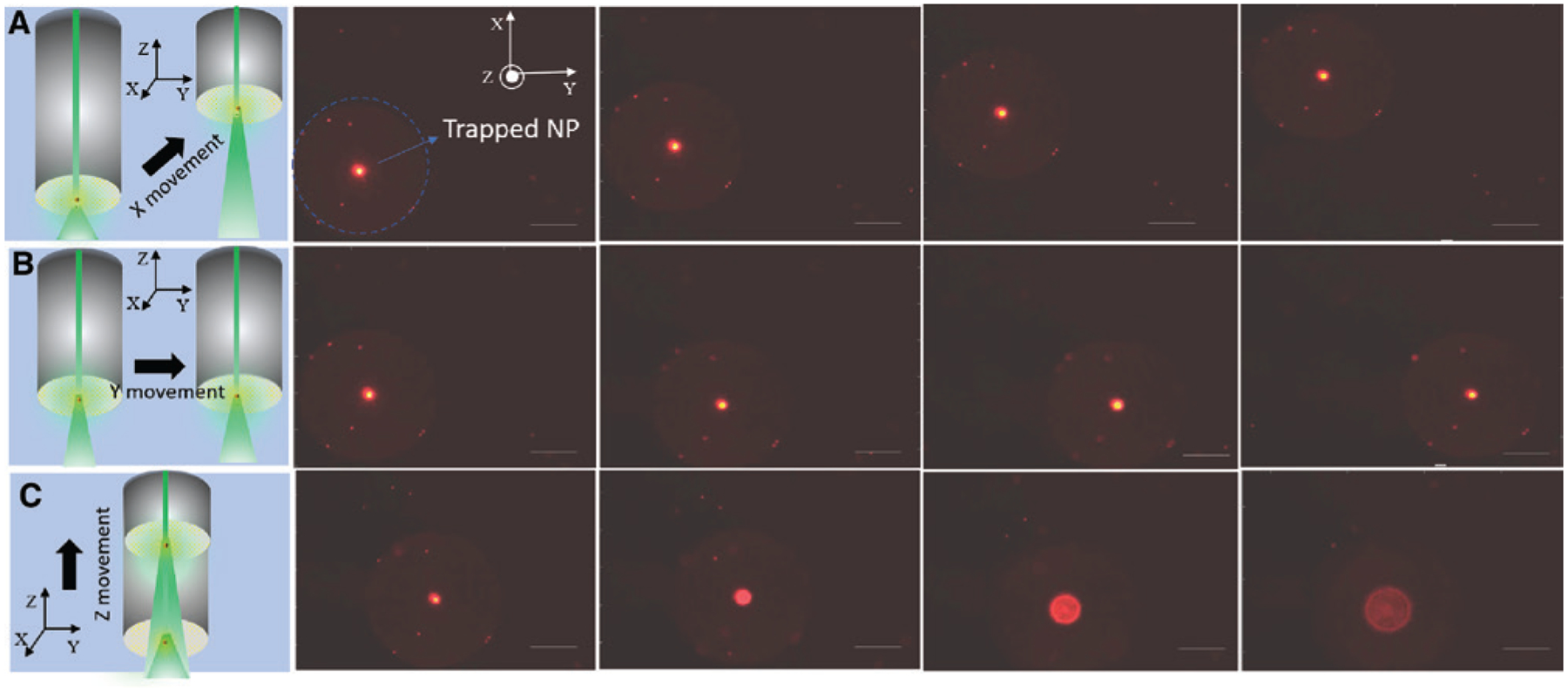
Three-dimensional manipulation of a single 200 nm fluorescent polystyrene nanoparticle in the (A) X direction, (B) Y direction, and (C) Z direction. Small, faint, stationary bright spots on the fiber surface correspond to some of the nanoparticles stuck at the cladding of the optical fiber tip. The scale bar for the figure is 50 μm.

The trapping and manipulation of particles using our previous opto-thermophoretic tweezers have been limited to in-plane, i.e. 2D [9]. The translation of the opto-thermophoretic tweezers to an optical fiber platform not only makes the whole system simple, cheap, robust, and easy to use but also provides an additional advantage of manipulating objects in three dimensions. Figure 1B shows a simple experimental setup to realize trapping and manipulation of nanoparticles in three dimensions. Briefly, a 532-nm diode-pumped solid-state laser (Genesis MX STM-1 W; Coherent) was coupled to OTFT using an optical fiber coupler (Thorlabs). The OTFT was attached to a three-axis manipulation stage (Nanomax 300, Thorlabs), with the OTFT tip immersed into the nanoparticle solution drop placed on a glass coverslip. It may be noted that OTFT could be placed in two different configurations, either normal to the glass coverslip (normal mode operation), as shown in Figure 1B, or parallel to the glass coverslip (parallel mode operation), as shown in the inset of Figure 1B. For parallel mode operation of OTFT, the arrangement in the red box shown in Figure 1B was replaced by the arrangement shown in the inset of Figure 1B. The selection of a particular configuration of OTFT for an experiment was based on the objective to achieve the best imaging and visualization of the concept to be demonstrated. The fiber tip was illuminated using a white light source and imaged using a 20× objective lens and a charge coupled device (Nikon) camera. Figure 2A–C shows the trapping of a single 200 nm fluorescent polystyrene nanoparticle at the core of the OTFT and its manipulation in three dimensions along the X, Y, and Z directions respectively. The output power from the fiber tip was measured to be 800 μW. The movement of the trapped particle in the Z direction is seen as the progressive defocusing of the image of the trapped nanoparticle as it moves away from the focus of the objective lens (see Supplementary Video 1 for real-time video). It may be noted that the particle manipulation resolution and range are limited only by the specifications of the mechanical stage on which the OTFT is mounted, which was 5 nm and 4 mm, respectively, in our case. Also, we observed that by using OTFT, the nanoparticle can be manipulated at a much faster speed compared to the conventional laser beam manipulation on 2D AuNI substrates. We attribute this to the fundamental design of the optical fiber used in OTFT, where the core of the fiber and the laser beam is always aligned and therefore the thermal hotspot at the core of OTFT (trapping position) is fixed, while the particle is manipulated indirectly by moving the fiber. This is not the case in conventional laser beam manipulation on 2D AuNI substrates, where the particle is manipulated directly by creating a new thermal hotspot each time on the AuNI substrate by moving the laser beam. In our current demonstration, the smallest particle size that was trapped and manipulated using OTFT was 100 nm Au nanoparticles. However, Au nanoparticles as small as 20 nm can be trapped using the opto-thermoelectric tweezers, as demonstrated in our previous work [9]. The size limit of the trapped particles can be further pushed down to sub-10 nm by increasing the temperature gradient through substrate optimization or use of an ultra-fast laser. The thermoelectric field responsible for trapping of nanoparticles is independent of the particle size. Once trapped, the particle could change the temperature distribution and thus change the electric field. Although our demonstration of 3D trapping and manipulation was shown for a single 200 nm polystyrene nanoparticle, OTFT can be readily extended to simultaneously manipulate multiple particles and even to manipulate particles of different compositions, shapes, and sizes, including metallic particles and biological cell (see Supplementary Information). The 3D manipulation of nanoparticles of diverse sizes and material using OTFT may open its use in several applications such as 3D assembly of nanoparticles [47] and cells [48], which is useful in biology, nano-manufacturing, and optical devices [49].

### 2.3 OTFT nanoparticle concentrator

**Figure 3:**
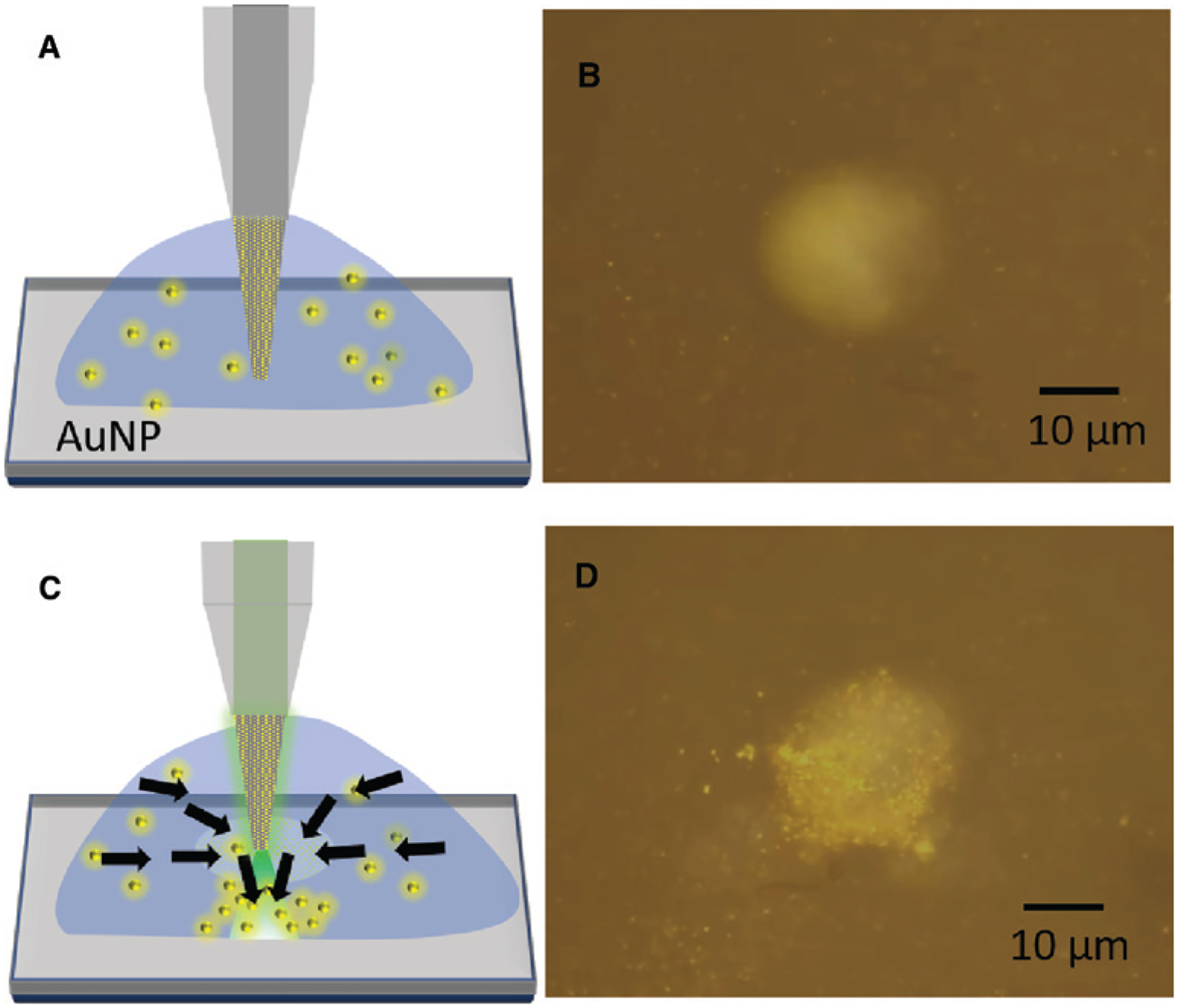
OTFT nanoparticle concentrator. (A) Schematic of the multimode tapered-OTFT immersed in the 200 nm Au nanoparticles (AuNP) solution. (B) Microscope image of the fabricated multimode tapered-OTFT (normal mode operation) immersed in 200 nm Au nanoparticle solution with no output optical power. The image is obtained by focusing the objective lens on the top surface of the glass coverslip with the tapered-OTFT tip placed 20 μm above the glass surface. (C) Schematic of the multimode tapered-OTFT with 10 mW of output power. The arrows demonstrate the flow of nanoparticles toward the tapered fiber due to the convective flow and then directed toward the glass surface. (D) Microscope image of the multimode tapered-OTFT showing the concentration of 200 nm Au nanoparticles on the glass surface 16 s after the laser is turned on.

OTFT can trap single and multiple nanoparticles using very low output power of less than 1 mW, as was shown in the previous section. In this optical power range, the thermoelectric force is the dominant force as compared to the convection forces or optical forces. However, by increasing the power output, the convection and optical forces can become significant. By simply regulating the power output of OTFT, various forces such as the thermophoretic forces, convection forces and optical forces can be judiciously combined on a single platform to achieve desired applications. One such application is the use of OTFT as a nanoparticle concentrator to concentrate nanoparticles spatially in a solution at the desired location. OTFT use both the strong thermal convective flow and thermophoretic forces to attract nanoparticles to the OTFT tip. After the particles approach close to the fiber tip, they are directed to the specific location by the strong optical scattering forces. The OTFT presented in the previous section can be directly used as particle concentrator by simply increasing the power launched from the fiber core. An increase in power results in an increase in the convective flow and thermophoretic forces pulling particles toward the fiber tip and at the same time directing them in the desired direction as they reach close to the OTFT tip. However, this process is not efficient as nanoparticles only from a limited region can be collected due to the weak convective flow. This is because only a small region, the core of the fiber (diameter = 9 μm), is heated. To improve the efficiency of OTFT as a nanoparticle concentrator, we modified the OTFT design. The modified OTFT is fabricated using a tapered multimode fiber instead of a flat-end SMF. This increases the thermoplasmonic area (AuNIs) exposed to the light propagating in the fiber core as optical power is leaked at the tapered end of OTFT, which has no cladding, as shown in Figure 3A. A multimode fiber with a core diameter of 50 μm was tapered down to 10 μm (see Methods section for tapering of optical fiber) close to the size of the core of a SMF. The large thermoplasmonic surface area exposed to light around the fiber core causes a strong convective flow, which draws particle from large distances toward the fiber tip while maintaining a strong temperature gradient at the fiber tip. Figure 3A shows the schematic of the OTFT nanoparticle concentrator immersed in the 200 nm Au nanoparticle solution. With no output power from the OTFT, the nanoparticles are randomly distributed in the droplet, as shown in Figure 3B. By launching a power of around 10 mW into the OTFT, the particles start to concentrate at the glass coverslip surface, where the tip of OTFT is positioned, as shown by the schematic in Figure 3C. The nanoparticles as far as 100 μm from the OTFT can be quickly drawn to the OTFT tip with high speeds. Figure 3D shows the concentrated nanoparticles at the glass surface 16 s after the laser is turned on (see Supplementary Video 2 for real-time video). A tapered fiber was employed for massive trapping and migration of particles using photophoretic and temperature gradient forces [50], which was applicable to the micron-sized dielectric particles. Our OTFT is different from the previous technique in terms of the working mechanism. With a combination of thermoelectric force, convection force, and optical scattering force, our OTFT can concentrate particles of variable compositions and sizes, including nanoscale metallic particles. OTFT concentrator can find applications in providing localized and site-specific concentration of nanoparticles in small volumes such as a single droplet.

### 2.4 OTFT nanopipette for single nanoparticle delivery

The study of nanoparticle-cell interactions at the single nanoparticle-cell level is an important area of research for understanding the fundamental molecular mechanism in cellular biology, drug delivery, etc. For example, the virus-cell interactions at single cell level can unravel the mechanisms of virus infectivity [51]. This requires manipulation tools that can precisely deliver nanoparticles to specific sites on the cell surface without damaging the cell or the nanoparticle of interest. Since opto-thermophoretic tweezers can trap and manipulate a broad variety of nanoparticles with a very low optical power, it can be an ideal tool for such applications. Recently, Au nanoparticles as small as 20 nm and liposomes were trapped and manipulated using opto-thermophoretic tweezers with less than 1 mW of laser power [9, 17], which are mostly used as carriers for drug discovery and delivery [52, 53]. In this section, we show OTFT as a nanopipette, which could be used for single particle delivery to the membrane of lipid vesicles/cells. Two different approaches were used to deliver nanoparticles to the specific site on the membrane of the lipid vesicles: direct and remote. We used 200 nm fluorescent polystyrene nanoparticle and 200 nm Au nanoparticle for delivery to a single large unilamellar lipid vesicle as a model system to demonstrate the two processes respectively. We chose two different types of nanoparticles only to show the versatility of our OTFT, but both the nanoparticles can be used for either of the delivery process.

**Figure 4:**
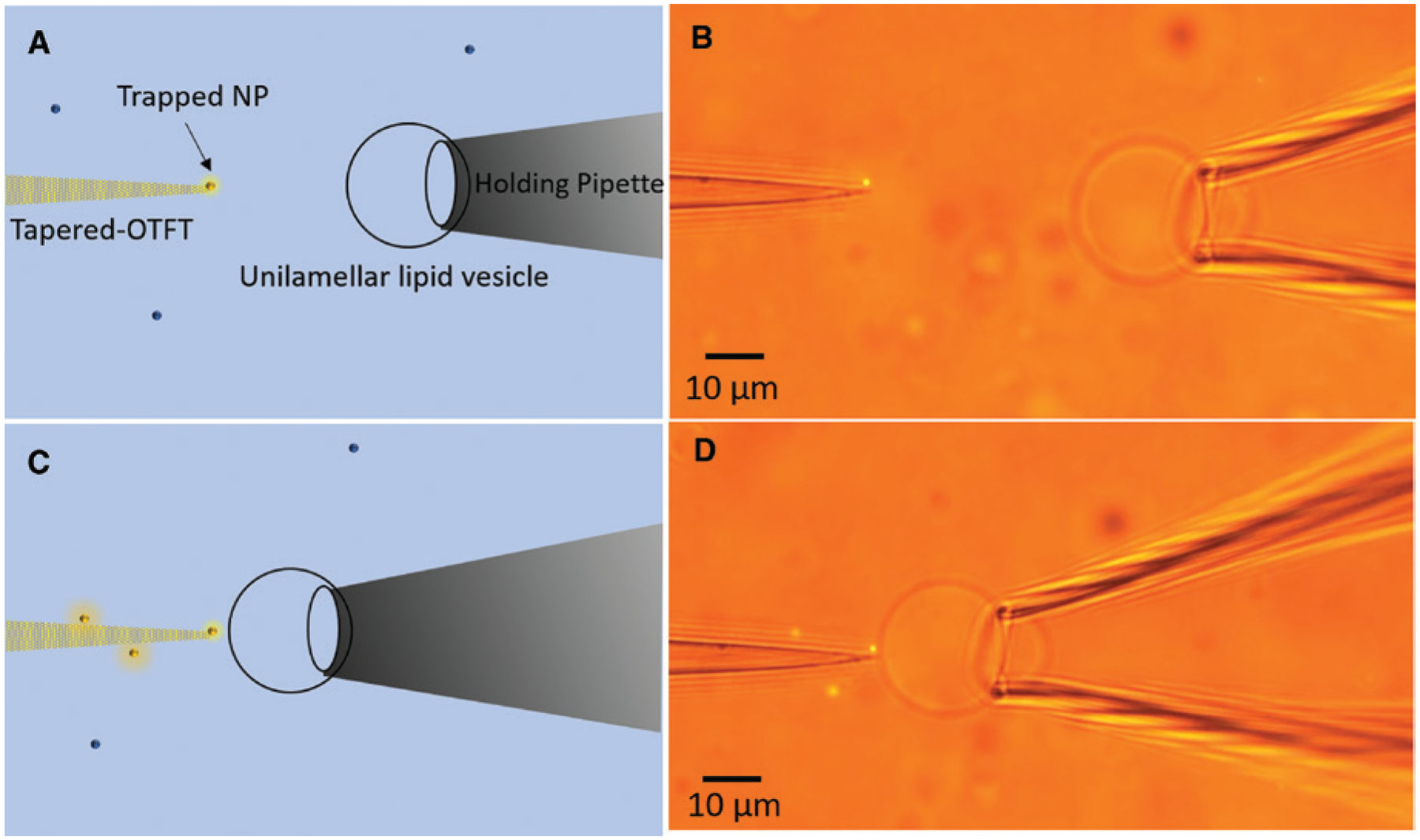
Direct delivery of nanoparticles. (A) Schematic of the tapered-OTFT nanopipette for direct single-particle delivery to a large unilamellar lipid vesicle. (B) Microscope image of tapered-OTFT nanopipette (parallel mode operation) holding a single 200 nm fluorescent polystyrene nanoparticle at the tip, ready for delivery. (C) Schematic of the tapered-OTFT nanopipette delivering a single 200 nm polystyrene nanoparticle to the lipid vesicle membrane. (D) Microscope image of tapered-OTFT nanopipette delivering a single 200 nm fluorescent polystyrene nanoparticle to the lipid vesicle membrane.

Figure 4A shows the schematic for the direct delivery process of a single nanoparticle to the lipid vesicle. A single nanoparticle is initially trapped at the tip of a tapered OTFT (see Methods section for fabrication details). We use a tapered OTFT instead of a flat-end OTFT to increase the spatial precision of particle delivery, but tapering of the fiber is not a necessary condition to achieve trapping and manipulation of the nanoparticle. Figure 4B shows the microscope image of the tapered OTFT with a single 200 nm fluorescent nanoparticle trapped at its tip. The tip of the OTFT is around 1 μm in diameter. A large unilamellar lipid vesicle was immobilized on the holding pipette (see Methods section for fabrication). Once the particle is trapped at the tip of a tapered OTFT, it can be delivered directly to the desired location on the membrane of the lipid vesicles as illustrated in Figure 4C. The OTFT nanopipette can precisely control the position of delivery and the spacing between the trapped nanoparticle and lipid vesicle. Figure 4D shows the delivery of 200 nm polystyrene nanoparticle to the lipid vesicle membrane (see Supplementary Video 3 for real-time video). The use of tapered-OTFT as a nanopipette would be particularly advantageous in applications where biological nanoparticles or nanoparticles coated with biomolecules need to be incubated near the cell membrane for long periods without damaging them or losing their biological activity because of its very low operational power. An interesting application would be the study of single exosome interactions with cells at single cell level to understand the mechanism of cargo transfer between them by endocytosis, fusion, etc. [54].

**Figure 5:**
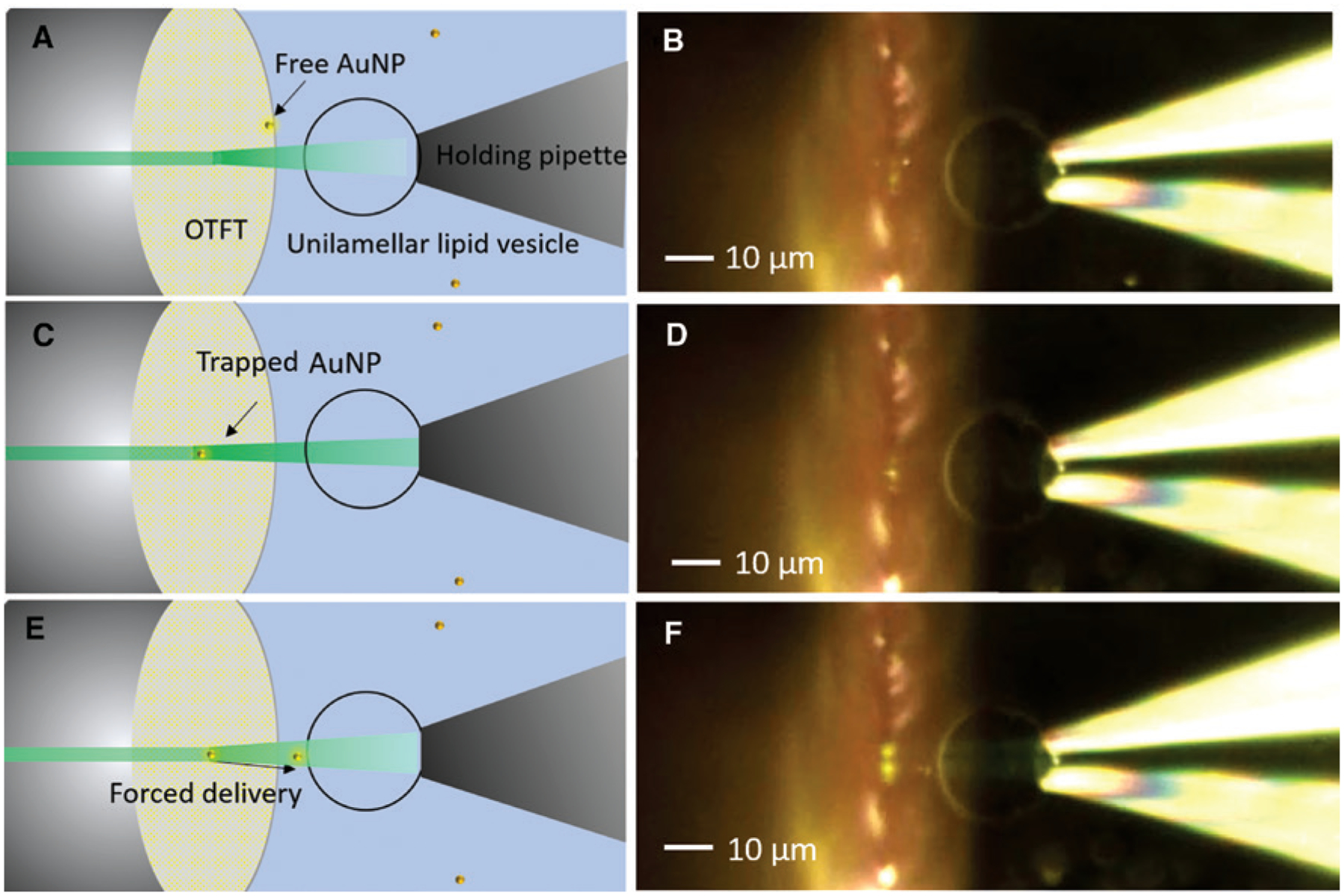
Remote delivery of nanoparticles. (A) Schematic showing the setup for remote delivery of nanoparticles to lipid vesicles using OTFT. (B) Microscope image showing the OTFT (parallel mode) along with the immobilized lipid vesicle with a freely diffusing 200 nm Au nanoparticle in the solution (C) Schematic showing the capture of Au nanoparticle using OTFT at low output power. (D) Microscope image showing the trapping of 200 nm Au nanoparticle using OTFT. (E) Schematic showing the forced delivery of nanoparticle using spiked output power from OTFT. (F) Microscope image showing the remote delivery of 200 nm Au nanoparticle to the lipid vesicle immobilized on the holding pipette.

Figures 5A, C, and E shows the schematic for the remote delivery of single nanoparticle to the lipid vesicle. A single nanoparticle was initially captured using the OTFT at low optical power and then aligned with the target lipid vesicle, which was immobilized on the holding pipette. The power output through the fiber was then spiked for a short duration (nearly 1–2 s), which launches the nanoparticle in the direction of the lipid vesicle due to the large optical scattering force. The position of the OTFT tip relative to the lipid vesicle and spiked optical power can be adjusted to control the position and force for nanoparticle delivery. Figure 5B shows the free 200 nm AuNP in the solution, which was captured by the OTFT using very low output power of around 800 μW, as shown in Figure 5D. The trapped AuNP was then remotely delivered to the membrane of the lipid vesicle positioned at a distance of 10 μm away from the Au nanoparticle at the velocity of 40 μm/sec by increasing the power output from the fiber to 5 mW (see Supplementary Video 4 for real-time video). The AuNP velocity was calculated from the time trajectory of the AuNP extracted from the real-time video.

### 2.5 OTFT nanoprobe

**Figure 6:**
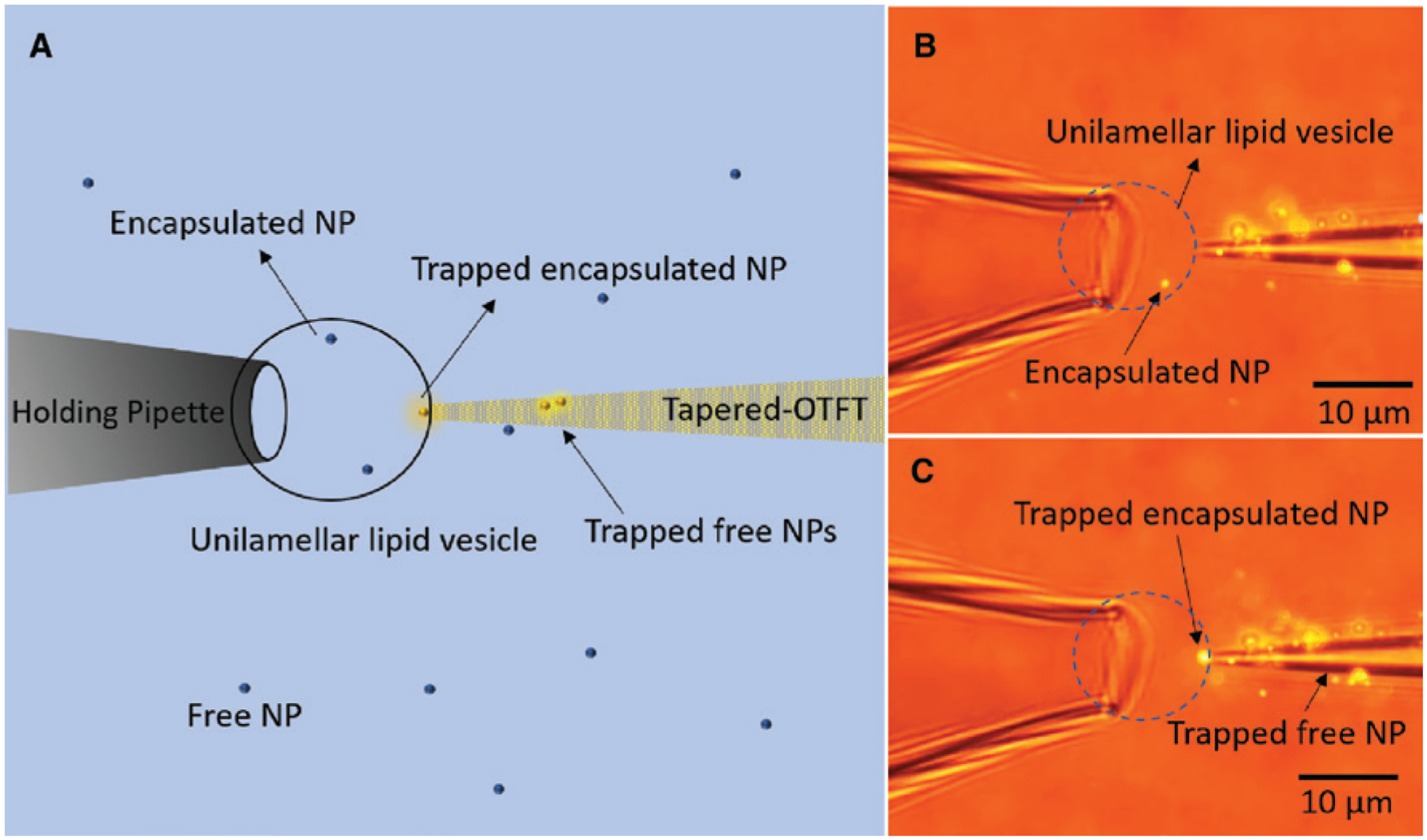
Trapping of nanoparticle encapsulated inside lipid vesicle. (A) Schematic of the tapered OTFT used for trapping 200 nm fluorescent nanoparticle encapsulated in a large unilamellar lipid vesicle. (B) Microscope image showing the tapered OTFT (parallel mode) in contact with the membrane of the lipid vesicle. The arrow pointing to the bright spot indicates the 200 nm fluorescent nanoparticle (NP) encapsulated inside the immobilized lipid vesicle. (C) Microscope image showing the trapping of encapsulated nanoparticle at the tip of the tapered OTFT.

The use of OTFT as a nanopipette for single particle delivery to cells shows its potential for applications in extracellular studies. However, for intracellular studies, OTFT should be able to trap particles inside the cell membrane. In this section, we demonstrate the capability of OTFT to act as nanoprobes for simultaneous trapping and sensing of nanoparticles inside a living cell. We used a large unilamellar lipid vesicle encapsulating a single 200 nm fluorescent polystyrene nanoparticle as a model system. The nanoparticle-encapsulated lipid vesicle was immobilized on a holding pipette by mechanical suction (see Methods section for the preparation of lipid vesicles encapsulated with nanoparticles). A tapered OTFT was used to trap the 200 nm fluorescent polystyrene nanoparticle, which was freely diffusing inside the lipid vesicles by bringing the tip of the fiber slowly in contact with the lipid membrane. Figure 6A shows the schematic of the trapping process. The output power of the tapered OTFT used for trapping was 800 μW. Figure 6B shows the microscope image of the lipid vesicle with a freely diffusing encapsulated 200 nm polystyrene nanoparticle immobilized on the glass pipette. After some time, the nanoparticle was trapped at the lipid vesicle membrane as shown in Figure 6C (see Supplementary Video 5 for real-time video). We do not insert the tapered OTFT tip inside the lipid vesicle, but this is possible by reducing the fiber tip size to less than 500 nm, in which case the particles could be trapped and manipulated inside the lipid vesicle rather than at the membrane interface. It should be noted that no CTAC was used in the experiment and the polystyrene nanoparticles were suspended in deionized (DI) water in the lipid vesicle. Thus, the trapping was based on the interfacial-entropy-driven thermophoresis as described in our earlier work [8]. Currently, the intracellular trapping is therefore limited to dielectric particles. To advance the intracellular trapping of metallic particles, we need biocompatible molecules that can form micelles with a positive Soret coefficient to generate thermoelectric force, which is an area we wish to explore in our future studies. A significant advantage of OTFT over other fiber tweezers when used as nanoprobes is the use of very low power for trapping, which would cause no damage to the cells or the cargo inside the cells during intracellular studies. The use of OTFT nanoprobe also provides the advantage of collecting the detected optical signal through the same fiber, making the whole system simple, efficient, and easier to operate. Some exciting applications that we envision for the future is the sensing of complex cargo such as proteins and RNA inside living cells by encapsulating Au nanoparticles coated with target-specific proteins or for single cell biopsies [55, 56].

## 3 Conclusion

We have developed OTFT for trapping and manipulation of nanoparticles in three dimensions. Furthermore, we demonstrated possible applications where OTFT could be particularly advantageous, such as in nanoparticle concentration and precise nanoparticle delivery to study nanoparticle-cell interactions at a single-cell level. We also showed how OTFT could be used as nanoprobes in cellular biology by trapping nanoparticles encapsulated within lipid vesicles as a model system. The use of CTAC for trapping and manipulation of nanoparticles might not be compatible for some biological applications, which would require replacement of CTAC with biocompatible molecules that could provide the thermoelectric effect in a temperature gradient. All these demonstrations would open applications of OTFT in various fields, such as assembly of nanoparticles and cell into 3D superstructures, study of exosome-cell interactions, and delivery and control of cargos within a living cell. With their simple design, easy fabrication, low-power operation, and versatility, OTFT would become a powerful tool for various applications in nanomanufacturing, nanomedicine, and life sciences.

## 4 Methods

### 4.1 Fabrication of OTFT and holding pipette

A commercially available single-mode pigtail fiber (core/cladding: 9/125 μm; Corning Inc.) was used to fabricate the OTFT. The flat cleaved end of the fiber pigtail was cleaned using isopropyl alcohol, followed by the deposition of 4.5-nm thin gold film at the tip of the fiber using Denton thermal evaporator; base pressure was 9 × 10^−6^ torr. The fiber tip was than annealed using a hot gun at 550°C for 2 h. This led to the formation of uniform AuNIs at the tip of the fiber. For fabrication of tapered-OTFT, the flat cleaved SMF was replaced by a tapered SMF in the above fabrication process. The tapered optical fiber is obtained by pulling a commercial SMF using a glass pipette puller (P-2000, Sutter Instrument Co.). The pipette puller parameters, such as heat, delay, and pull force, were optimized to fabricate fiber with a gradual taper and desired tip size. The smallest tapered fiber tip that was fabricated was 1 μm. The holding pipette used for immobilization of the lipid vesicle was fabricated using a borosilicate glass capillary with an inner diameter of 0.75 mm and outer diameter of 1 mm (B100-75-10, Sutter Instrument Co.). The glass capillary was pulled using the glass pipette puller to form a tapered opening with a size of 8–10 μm. The other end of the capillary was connected to a PE10 tubing and a syringe for mechanical suction to immobilize the large unilamellar lipid vesicle.

### 4.2 Preparation of lipid vesicles and nanoparticle solutions

1,2-Dioleoyl-*sn*-glycero-3-phosphocholine (DOPC) and 1,2-dioleoyl-*sn*-glycero-3-phospho-(1′-rac-glycerol) (DOPG) sodium salt powders, obtained from Avanti Polar Lipids, were dissolved in chloroform, CH_3_Cl (50 mg/5 ml), in brown glass vials with Teflon-coated caps and stored under N_2_ atmosphere at −20 °C. After, 100 μl of DOPC:DOPG (4:1) lipid solution in chloroform was deposited into a glass vial and dried under N_2_ for 10 min to form a dried lipid film and placed under vacuum for 2 h. Thereafter, the lipid film was rehydrated with 2 ml of N_2_-degassed water (resistivity of 18.2 mΩ cm) and stirred with a magnetic stir bar at 1100 rpm for 1 h. Unilamellar lipid vesicles of different sizes were formed, which were than extracted and further diluted for use in the experiments. For the preparation of lipid vesicles encapsulated with 200 nm fluorescent polystyrene nanoparticles (excitation/emission: 540/600 nm), the dried lipid film was rehydrated with 1 ml of 200 nm fluorescent polystyrene nanoparticle solution in DI water with a concentration of 0.1% w/v. The 200 nm polystyrene nanoparticles get encapsulated randomly inside large unilamellar lipid vesicles during the above process. It may be noted that the encapsulation process is not well controlled and therefore results in the formation of lipid vesicles with varied number of encapsulated nanoparticles ranging from zero to hundreds. Large unilamellar lipid vesicles with a single 200 nm polystyrene nanoparticle encapsulation were used in the experiments for visual clarity.

The 200 nm fluorescent polystyrene nanoparticle solutions (excitation/emission wavelengths: 540/600 nm) used in the experiments were prepared by diluting the as-purchased solutions (Bang Laboratories) in 2 mm CTAC to reach a final concentration of 1 × 10^7^ particles/ml. The 200 nm Au nanoparticles solutions were prepared by diluting the as-purchased solutions (NanoPartz, Inc.) in 2 mm CTAC to reach a final concentration of 1 × 10^7^ particles/ml.

## Supplementary Material

supplementary notes and videos

## References

[1] Braun M, Cichos F. Optically controlled thermophoretic trapping of single nano-objects. ACS Nano 2013;7:11200–8. 24215133 10.1021/nn404980k

[2] Braun M, Würger A, Cichos F. Trapping of single nano-objects in dynamic temperature fields. Phys Chem Chem Phys 2014;16:15207–13. 24939651 10.1039/c4cp01560f

[3] Braun D, Libchaber A. Trapping of DNA by thermophoretic depletion and convection. Phys Rev Lett 2002;89:2–5. 10.1103/PhysRevLett.89.188103 12398641

[4] Piazza R Thermophoresis: moving particles with thermal gradients. Soft Matter 2008;4:1740–4.

[5] Braun M, Bregulla AP, Günther K, Mertig M, Cichos F. Single molecules trapped by dynamic inhomogeneous temperature fields. Nano Lett 2015;15:5499–505. 26161841 10.1021/acs.nanolett.5b01999

[6] Lin L, Hill EH, Peng X, Zheng Y. Optothermal manipulations of colloidal particles and living cells. Acc Chem Res 2018;51:1465–74. 29799720 10.1021/acs.accounts.8b00102 PMC6008228

[7] Li J, Lin L, Inoue Y, Zheng Y. Opto-thermophoretic tweezers and assembly. J Micro Nano-Manufacturing 2018;6:1–10. 10.1115/1.4041615 PMC8597552 35832388

[8] Lin L, Peng X, Mao Z, Wei X, Xie C, Zheng Y. Interfacial-entropy-driven thermophoretic tweezers. Lab Chip 2017;17:3061–70. 28805878 10.1039/c7lc00432j

[9] Lin L, Wang M, Peng X, Opto-thermoelectric nanotweezers. Nat Photonics 2018;12:195–201. 29785202 10.1038/s41566-018-0134-3 PMC5958900

[10] Liu Y, Lin L, Bangalore Rajeeva B, Nanoradiator-mediated deterministic opto-thermoelectric manipulation. ACS Nano 2018;12:10383–92. 30226980 10.1021/acsnano.8b05824 PMC6232078

[11] Yoo D, Gurunatha KL, Choi HK, Low-power optical trapping of nanoparticles and proteins with resonant coaxial nanoaperture using 10 nm gap. Nano Lett 2018;18:3637–42. 29763566 10.1021/acs.nanolett.8b00732

[12] Zhao Y, Saleh AAE, Dionne JA. Enantioselective optical trapping of chiral nanoparticles with plasmonic tweezers. ACS Photonics 2016;3:304–9.

[13] Galloway CM, Kreuzer MP, Aćimović SS, Plasmon-assisted delivery of single nano-objects in an optical hot spot. Nano Lett 2013;13:4299–304. 23915079 10.1021/nl402071p

[14] Neuman KC, Block SM. Optical trapping. Rev Sci Instrum 2004;75:2787–809. 16878180 10.1063/1.1785844 PMC1523313

[15] Nieminen TA, Knöner G, Heckenberg NR, Rubinsztein-Dunlop H. Physics of optical tweezers. In: Berns MW, Greulich KO, eds. Laser manipulation of cells and tissues. Vol. 82. San Diego, USA, Academic Press, 2007; pp. 207–36. 10.1016/S0091-679X(06)82006-6 17586258

[16] Lin L, Peng X, Wei X, Mao Z, Xie C, Zheng Y. Thermophoretic tweezers for low-power and versatile manipulation of biological cells. ACS Nano 2017;11:3147–54. 28230355 10.1021/acsnano.7b00207

[17] Hill EH, Li J, Lin L, Liu Y, Zheng Y. Opto-thermophoretic attraction, trapping, and dynamic manipula-tion of lipid vesicles. Langmuir 2018;34:13252–62. 30350700 10.1021/acs.langmuir.8b01979 PMC6246038

[18] Kang Z, Chen J, Wu SY, Trapping and assembling of particles and live cells on large-scale random gold nano-island substrates. Sci Rep 2015;5:9978. 25928045 10.1038/srep09978 PMC5386207

[19] Chen J, Kang Z, Kong SK, Ho HP. Plasmonic random nanostructures on fiber tip for trapping live cells and colloidal particles. Opt Lett 2015;40:3926. 26368677 10.1364/OL.40.003926

[20] Flores-Flores E, Torres-Hurtado SA, Páez R, Trapping and manipulation of microparticles using laser-induced convection currents and photophoresis. Biomed Opt Express 2015;6:4079. 26504655 10.1364/BOE.6.004079 PMC4605064

[21] Chen J, Cong H, Loo FC, Thermal gradient induced tweezers for the manipulation of particles and cells. Sci. Rep 2016;6:1–13. 27853191 10.1038/srep35814 PMC5113121

[22] Smith CLC, Thilsted AH, Pedersen JN, Photothermal transport of DNA in entropy-landscape plasmonic waveguides. ACS Nano 2017;11:4553–63. 28453288 10.1021/acsnano.6b08563

[23] Thamdrup LH, Larsen NB, Kristensen A. Light-induced local heating for thermophoretic manipulation of DNA in polymer micro- And nanochannels. Nano Lett 2010;10:826–32. 20166745 10.1021/nl903190q

[24] Ndukaife JC, Mishra A, Guler U, Nnanna AGA, Wereley ST, Boltasseva A. Photothermal heating enabled by plasmonic nanostructures for electrokinetic manipulation and sorting of particles. ACS Nano 2014;8:9035–43. 25144369 10.1021/nn502294w

[25] Lin L, Zhang J, Peng X, Opto-thermophoretic assembly of colloidal matter. Sci Adv 2017;3:1–10. 10.1126/sciadv.1700458 PMC5590781 28913423

[26] Peng X, Li J, Lin L, Liu Y, Zheng Y. Opto-thermophoretic manipulation and construction of colloidal superstructures in photocurable hydrogels. ACS Appl Nano Mater 2018;1:acsanm.8b00766. 10.1021/acsanm.8b00766 PMC6516762 31106296

[27] Lin L, Peng X, Zheng Y. Reconfigurable opto-thermoelectric printing of colloidal particles. Chem Commun 2017;53:7357–60. 10.1039/c7cc03530f 28569897

[28] Ribeiro RSR, Soppera O, Oliva AG, Guerreiro A, Jorge PAS. New trends on optical fiber tweezers. J Light Technol 2015;33:3394–405.

[29] Xin H, Li B. Fiber-based optical trapping and manipulation. Front Optoelectron 2017. 10.1007/s12200-017-0755-z.

[30] Xin H, Li B. Optical orientation and shifting of a single multi-walled carbon nanotube. Light Sci Appl 2014;3:e205.

[31] Xin H, Li Y, Li B. Cell patterning: controllable patterning of different cells via optical assembly of 1D periodic cell structures (Adv. Funct. Mater. 19/2015). Adv Funct Mater 2015;25:2786.

[32] Xin H, Li Y, Xu D, Zhang Y, Chen C-H, Li B. Single upconversion nanoparticle-bacterium cotrapping for single-bacterium labeling and analysis. Small 2017;13:1603418. 10.1002/smll.201603418 28092436

[33] Xin H, Li Y, Liu X, Li B. Escherichia coli-based biophotonic waveguides. Nano Lett 2013;13:3408–13. 23786313 10.1021/nl401870d

[34] Xin H, Li Y, Li B. Bacteria-based branched structures for bionanophotonics. Laser Photon Rev 2015;9:554–63.

[35] Liu Z, Guo C, Yang J, Yuan L. Tapered fiber optical tweezers for microscopic particle trapping: fabrication and application. Opt Express 2006;14:12510–6. 19529686 10.1364/oe.14.012510

[36] Xin H, Xu R, Li B. Optical trapping, driving, and arrangement of particles using a tapered fibre probe. Sci Rep 2012;2:818. 23150782 10.1038/srep00818 PMC3495291

[37] Mohanty KS, Liberale C, Mohanty SK, Degiorgio V. In depth fiber optic trapping of low-index microscopic objects. Appl Phys Lett 2008;92:151113.

[38] Ribeiro R, Queirós R, Soppera O, Guerreiro A, Jorge P. Optical fiber tweezers fabricated by guided wave photo-polymerization. Photonics 2015;2:634–45.

[39] Lyons ER, Sonek GJ. Confinement and bistability in a tapered hemispherically lensed optical fiber trap. Appl Phys Lett 1995;66:1584–6.

[40] Constable A, Kim J, Mervis J, Zarinetchi F, Prentiss M. Demonstration of a-fiber-optical light-force trap. Opt Lett 1993;18:1867–9. 19829431 10.1364/ol.18.001867

[41] Gelfand RM, Wheaton S, Gordon R. Cleaved fiber optic double nanohole optical tweezers for trapping nanoparticles. Opt Lett 2014;39:6415. 25490482 10.1364/OL.39.006415

[42] Berthelot J, Acimovic SS, Juan ML, Kreuzer MP, Renger J, Quidant R. Three-dimensional manipulation with scanning near-field optical nanotweezers. Nat Nanotechnol 2014;9:295–9. 24584272 10.1038/nnano.2014.24

[43] Saleh AAE, Sheikhoelislami S, Gastelum S, Dionne JA. Grating-flanked plasmonic coaxial apertures for efficient fiber optical tweezers. Opt Express 2016;24:20593. 27607663 10.1364/OE.24.020593

[44] Nikoobakht B, El-Sayed MA. Evidence for bilayer assembly of cationic surfactants on the surface of gold nanorods. Langmuir 2001;17:6368–74.

[45] Reichl M, Herzog M, Götz A, Braun D. Why charged molecules move across a temperature gradient: The role of electric fields. Phys Rev Lett 2014;112:1–5. 10.1103/PhysRevLett.112.198101 24877967

[46] Lin L, Peng X, Wang M, Light-directed reversible assembly of plasmonic nanoparticles using plasmon-enhanced thermophoresis. ACS Nano 2016;10:9659–68. 27640212 10.1021/acsnano.6b05486

[47] Sinclair G, Jordan P, Courtial J, Padgett M, Cooper J, Laczik ZJ. Assembly of 3-dimensional structures using programmable holographic optical tweezers. Opt Express 2004;12:5475. 19484108 10.1364/opex.12.005475

[48] Yoshida A, Tsuji S, Taniguchi H, Kenmotsu T, Sadakane K, Yoshikawa K. Manipulating living cells to construct a 3D single-cell assembly without an artificial scaffold. Polymers (Basel) 2017;9:1–10. 10.3390/polym9080319 PMC6418816 30970994

[49] Grzelczak M, Liz-Marzán LM. Colloidal nanoplasmonics: from building blocks to sensing devices. Langmuir 2013;29:4652–63. 23421758 10.1021/la4001544

[50] Xin H, Li X, Li B. Massive photothermal trapping and migration of particles by a tapered optical fiber. Opt Express 2011;19:17065. 21935067 10.1364/OE.19.017065

[51] Hou X, DeSantis MC, Tian C, Cheng W. Optical manipulation of a single human virus for study of viral-cell interactions. Proc SPIE Int Soc Opt Eng 2016:9922. pii: 992212. 27746582 10.1117/12.2239051 PMC5058341

[52] Kong FY, Zhang JW, Li RF, Wang ZX, Wang WJ, Wang W. Unique roles of gold nanoparticles in drug delivery, targeting and imaging applications. Molecules 2017;22:1445. 10.3390/molecules22091445 PMC6151763 28858253

[53] Allen TM, Cullis PR. Liposomal drug delivery systems: from concept to clinical applications. Adv Drug Deliv Rev 2013;65:36–48. 23036225 10.1016/j.addr.2012.09.037

[54] Prada I, Amin L, Furlan R, Legname G, Verderio C, Cojoc D. A new approach to follow a single extracellular vesicle-cell interaction using optical tweezers. Biotechniques 2016;60:35–41. 26757810 10.2144/000114371

[55] Cui Y, Irudayaraj J. Inside single cells: quantitative analysis with advanced optics and nanomaterials. Wiley Interdiscip Rev Nanomed Nanobiotechnol 2015;7:387–407. 25430077 10.1002/wnan.1321 PMC4397143

[56] Nadappuram BP, Cadinu P, Barik A, Nanoscale tweezers for single-cell biopsies. Nat Nanotechnol 2018;14:80–8. 30510280 10.1038/s41565-018-0315-8

