# Supplementary material for: Opto-thermophoretic fiber tweezers
Source: Nanophotonics. Author manuscript; Available in PMC 2021 Jul 20. (PMC8291374; doi:10.1515/nanoph-2018-0226)
Supplement: supplementary notes and videos [file NIHMS1625974-supplement-supplementary_notes_and_videos.zip › Supplement/Supplementary Information.docx]

**Supplementary Information**

Opto-thermophoretic fiber tweezers

Abhay Kotnala and Yuebing Zheng*

Department of Mechanical Engineering, Materials Science & Engineering Program and Texas Materials Institute, The University of Texas at Austin, Austin, TX 78712

*Corresponding author: [zheng@austin.utexas.edu](mailto:zheng@austin.utexas.edu)

1. Trapping of single 200 nm Au nanoparticle using OTFT

We used opto-thermophoretic fiber tweezers (OTFT) to trap a single 200 nm Au nanoparticle as shown in Figure S1. OTFT was used in the parallel mode and dark-field imaging was used to visualize the trapping of a single 200 nm Au nanoparticle. The 200 nm Au nanoparticle solution had a concentration of 1 × 10^7^ particles/ml and was prepared in 2mM CTAC.


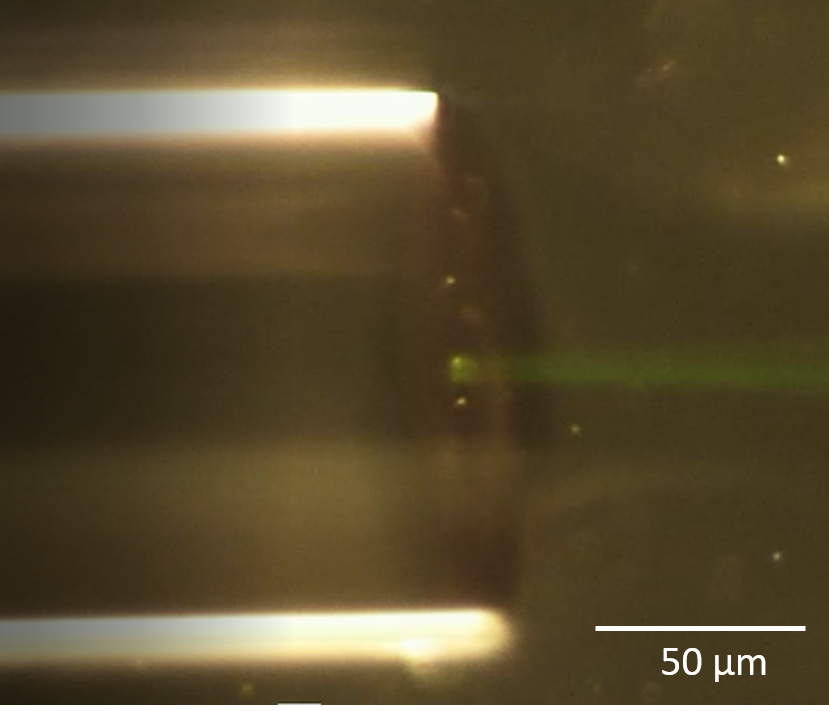


Figure S1: Trapping of a single 200 nm Au nanoparticle using OTFT. Near the fiber surface, the bright spot at the core of the fiber shows the trapped particle. The other bright spots outside the core of the fiber are the particles stuck at the fiber surface.

1. Trapping of single and multiple 500 nm polystyrene nanoparticles using OTFT

We used OTFT to trap a single 500 nm fluorescent polystyrene nanoparticle (excitation/emission wavelengths: 480/520 nm) as shown in Figure S2a. OTFT was used in the parallel mode and fluorescence imaging was used to visualize the trapping of a single 500 nm polystyrene nanoparticle. The 500 nm polystyrene nanoparticle solution had a concentration of 1 × 10^7^ particles/ml and was prepared in 2mM CTAC.

When the laser was on for the longer time, multiple particles were trapped at the core of the OTFT. Figure S2b shows the trapping of five 500 nm fluorescent polystyrene particles. The presence of multiple particles was confirmed by the increase in the fluorescence intensity at the core of the OTFT compared to the trapping of a single nanoparticle shown in figure S2a.


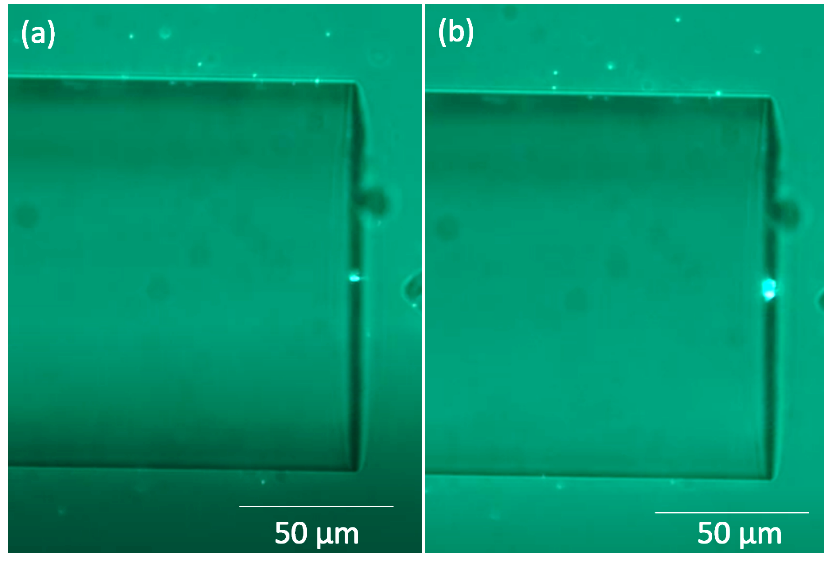


Figure S2 : (a) Trapping of a single 500 nm fluorescent polystyrene nanoparticle using OTFT. (b) Trapping of five 500 nm polystyrene nanoparticles using OTFT.

1. Trapping of single and multiple yeast cells using OTFT

OTFT was used to trap a single yeast cell as shown in Figure S3a. OTFT was used in the normal mode and bright-field imaging was used to visualize the trapping of a single yeast cell. The yeast cells were dispersed in DI water with a cell concentration of∼1 × 10^3^ cells/mm^3^.It may be noted that no CTAC was added in this case and the trapping was realized by the interfacial-entropy-driven opto-thermophoretic forces as described in our previous work ^1,2^.

When the laser was on for the longer time, multiple yeast cells were trapped at the core of the OTFT. Figure S3b shows the trapping of three yeast cells. Once trapped, the cells could be manipulated in 3D by moving the OTFT using the 3-axis manipulation stage.


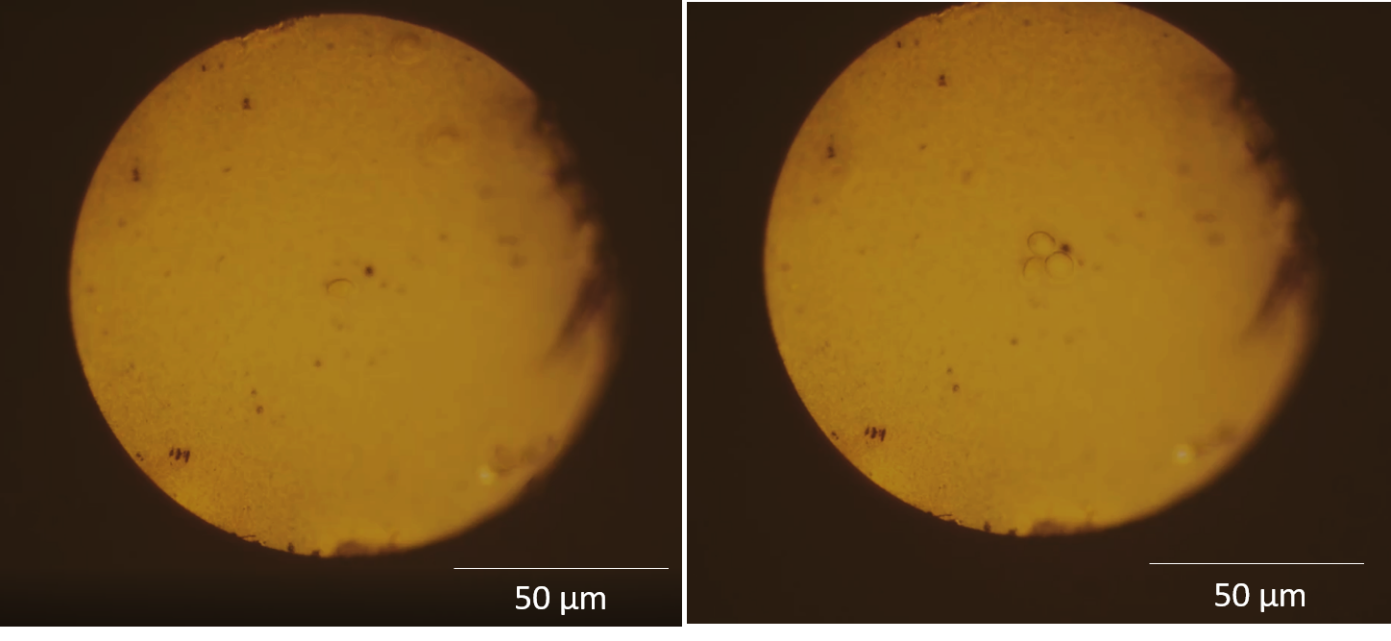


Figure S3 (a) Trapping of a single yeast cell using OTFT. (b) Trapping of three yeast cells using OTFT.

References

1. Lin L, Peng X, Mao Z, Wei X, Xie C, Zheng Y. Interfacial-entropy-driven thermophoretic tweezers. Lab Chip 2017, 17(18), 3061–70.

2. Lin L, Peng X, Wei X, Mao Z, Xie C, Zheng Y. Thermophoretic Tweezers for Low-Power and Versatile Manipulation of Biological Cells. ACS Nano 2017, 11(3), 3147–54.

Supplemental Material

To support the work, a supplementary document and five video clips are enclosed as supplemental material.

Supplementary video 1: Real-time video showing the 3D manipulation of single 200 nm fluorescent polystyrene nanoparticle using OTFT.

Supplementary video 2: Real-time video showing the concentration of 200 nm Au nanoparticles using tapered-OTFT.

Supplementary video 3: Real-time video showing the direct delivery of single 200 nm fluorescent nanoparticle to a lipid vesicle membrane using tapered-OTFT.

Supplementary video 4: Real-time video showing the remote delivery of a single 200 nm Au nanoparticle to a lipid vesicle membrane using OTFT.

Supplementary video 5: Real-time video showing the trapping of a single 200 fluorescent polystyrene nanoparticle encapsulated in a lipid vesicle using tapered-OTFT.
